# Utilidade da Idade, Creatinina e Fração de Ejeção - Modificação da Dieta no Escore de Doença Renal para Prever a Sobrevivência em Pacientes com Insuficiência Cardíaca

**DOI:** 10.36660/abc.20230158

**Published:** 2023-12-14

**Authors:** Rengin Çetin Güvenç, Tolga Sinan Güvenç, Yüksel Çavuşoğlu, Ahmet Temizhan, Mehmet Birhan Yılmaz

**Affiliations:** 1 Okan University Faculty of Medicine Department of Cardiology Istanbul Turquia Okan University Faculty of Medicine , Department of Cardiology , Istanbul – Turquia; 2 Istinye University Faculty of Medicine Department of Cardiology Istanbul Turquia Istinye University Faculty of Medicine , Department of Cardiology , Istanbul – Turquia; 3 Eskisehir Osmangazi University Department of Cardiology Eskisehir Turquia Eskisehir Osmangazi University , Department of Cardiology , Eskisehir – Turquia; 4 Ankara City Hospital Department of Cardiology Ankara Turquia Ankara City Hospital , Department of Cardiology , Ankara – Turquia; 5 Dokuz Eylul University Faculty of Medicine Department of Cardiology Izmir Turquia Dokuz Eylul University Faculty of Medicine , Department of Cardiology , Izmir – Turquia

**Keywords:** Insuficiência Cardíaca, Mortalidade, Sobrevivência

## Abstract

**Fundamento:**

Embora muitos modelos de risco tenham sido desenvolvidos para prever o prognóstico na insuficiência cardíaca (IC), esses modelos raramente são úteis para o clínico, pois incluem múltiplas variáveis que podem ser demoradas para serem obtidas, são geralmente difíceis de calcular e podem sofrer de *overfitting* estatístico.

**Objetivos:**

Investigar se um modelo mais simples, nomeadamente o escore ACEF-MDRD, poderia ser usado para prever a mortalidade em um ano em pacientes com IC.

**Métodos:**

748 casos do registro SELFIE-HF tinham dados completos para calcular o escore ACEF-MDRD. Os pacientes foram agrupados em tercis para análise. Para todos os testes, um valor de p <0,05 foi aceito como significativo.

**Resultados:**

Significativamente mais pacientes dentro do tercil ACEF-MDRD _alto_ (30,0%) morreram dentro de um ano, em comparação com outros tercis (10,8% e 16,1%, respectivamente, para ACEF-MDRD _baixo_ e ACEF-MDRD _med_ , p<0,001 para ambas as comparações). Houve uma diminuição gradual na sobrevida em um ano à medida que o escore ACEF-MDRD aumentou (log-rank p<0,001). ACEF-MDRD foi preditor independente de sobrevida após ajuste para outras variáveis (OR: 1,14, IC95%:1,04 – 1,24, p=0,006). O escore ACEF-MDRD ofereceu precisão semelhante ao escore GWTG-HF para prever a mortalidade em um ano (p=0,14).

**Conclusões:**

ACEF-MDRD é um preditor de mortalidade em pacientes com IC e sua utilidade é comparável a modelos semelhantes, porém mais complicados.

## Graphical abstract

**Figura Central:**
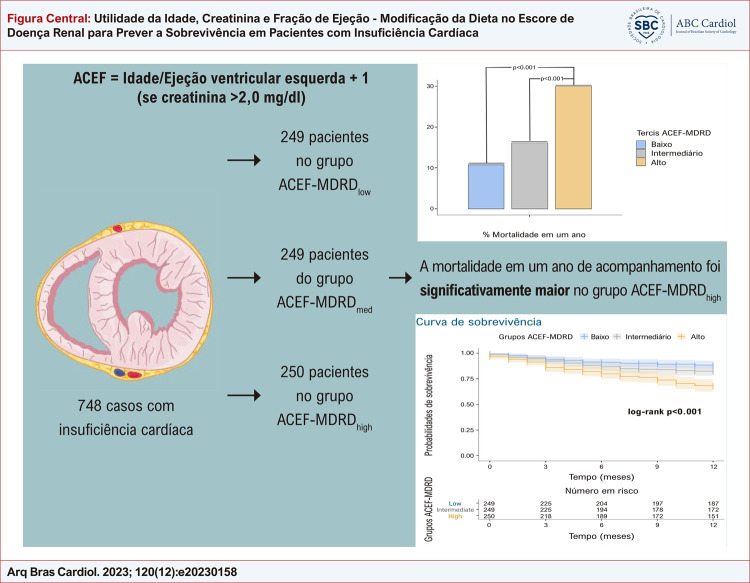
: Utilidade da Idade, Creatinina e Fração de Ejeção - Modificação da Dieta no Escore de Doença Renal para Prever a Sobrevivência em Pacientes com Insuficiência Cardíaca

Resumo do desenho do estudo e principais conclusões. ACEF: Idade, creatinina e fração de ejeção (Age, creatinine and ejection fraction) MDRD: Dieta Modificada em Doença Renal (Modified Diet in Renal Disease).

## Introdução

Estima-se que pelo menos 23 milhões de pessoas tenham insuficiência cardíaca (IC), tornando-a uma das doenças cardiovasculares mais comuns na era contemporânea. ^1^ Apesar dos avanços no rastreio, diagnóstico e tratamento da IC, as taxas de mortalidade permanecem elevadas, com uma taxa de 121 por 1.000 pacientes-ano para pacientes com fração de ejeção preservada (ICFEp) e 141 por 1.000 pacientes para pacientes com fração de ejeção reduzida (ICFEr). ^2^ Embora o julgamento clínico e os parâmetros individuais sejam comumente empregados para prognóstico, múltiplos modelos de risco também estão disponíveis para estimar a mortalidade e orientar decisões de manejo. ^3 - 7^

Um problema comum com estes modelos de risco é que eles geralmente sofrem de “ *overfitting* ” de múltiplas variáveis redundantes que não são úteis na estimativa do prognóstico em outras coortes de IC onde o a taxa de mortalidade difere da coorte de derivação original. ^8^ Além disso, a necessidade de usar inúmeras variáveis (e às vezes trabalhosas de obter) para calcular um único escore de risco para cada paciente com IC geralmente torna esses escores impraticáveis para uso clínico em uma clínica movimentada.

O escore de idade, creatinina e fração de ejeção (ACEF) foi inicialmente desenvolvido para prever a mortalidade pós-operatória após cirurgia cardiovascular, mantendo a “lei da parcimônia” em mente. ^8^ No entanto, estudos posteriores encontraram o escore ACEF ou suas modificações simples calculadas pela substituição da creatinina com taxa de filtração glomerular (TFG) com a equação Dieta Modificada em Doença Renal (MDRD) - o escore ACEF-MDRD - foram úteis para prever mortalidade ou complicações após intervenções coronárias percutâneas ou estruturais, bem como aqueles que apresentavam síndromes coronarianas agudas. ^9 - 12^ As variáveis individuais utilizadas para calcular o escore ACEF já foram demonstradas como preditoras de hospitalizações e mortalidade em pacientes com IC, e é razoável considerar que um escore calculado usando essas variáveis teria melhor utilidade na predição de mortalidade em IC. ^13 - 16^ Na presente análise, procuramos investigar se o escore ACEF-MDRD poderia prever a mortalidade em um ano em pacientes com IC e entender como o escore ACEF-MDRD se compara a outros modelos estabelecidos, porém mais complexos, como o Get With The Guidelines – Heart Failure – Siga as Diretrizes - de Insuficiência Cardíaca (GWTG-HF).

## Métodos

A concepção e execução do registo SELFIE-TR já foram publicadas anteriormente. ^17^ Para resumir, 23 centros de estudo representando todas as áreas geográficas da Turquia foram incluídos no estudo SELFIE-TR. O diagnóstico de IC foi estabelecido por meio de uma combinação de avaliação clínica, ecocardiográfica e laboratorial, e o diagnóstico foi confirmado de forma independente por pelo menos dois cardiologistas que trabalham em cada centro de estudo. Todos os pacientes com 18 anos ou mais e que aceitaram inscrição no estudo foram incluídos; nenhum critério de exclusão foi utilizado. Foram incluídos mil e cinquenta e quatro pacientes, e recentemente foram disponibilizados dados de sobrevida em um ano para 1.022 desses 1.054 pacientes. ^18^ Desses pacientes, 748 tinham dados completos para calcular o escore ACEF-MDRD, e todas as análises foram feitas usando esses registros.

Todos os pacientes do registro SELFIE-TR deram seu consentimento informado antes da inclusão, e o presente estudo foi conduzido de acordo com os princípios descritos na Declaração de Helsinque de 1975 e suas revisões. O estudo foi aprovado por um comitê de ética (aprovação nº 288-AU/003), e a aprovação regulatória foi obtida em cada centro de estudos de acordo com leis e outros regulamentos.

Todas as medições laboratoriais foram feitas nos centros individuais e as amostras utilizadas para análises foram retiradas logo após a inclusão do paciente no estudo. Nem todas as medidas estavam disponíveis para todos os pacientes devido às diferenças entre os centros em relação aos recursos locais. A taxa de filtração glomerular foi calculada usando a equação MDRD. A fração de ejeção foi medida com ecocardiografia bidimensional em cada centro de estudo com o método de Simpson modificado por dois cardiologistas cegos para a medição um do outro, e uma média dessas duas medições foi tomada como resultado final. O escore ACEF-MDRD foi calculado da seguinte forma:

Idade/fração de ejeção + 1 ponto para cada redução de 10 mL/min na TFG quando a TFG estava abaixo de 60 ml/m ^2^ /min.

### Análise estatística

O tamanho da amostra foi determinado pelo número de casos elegíveis para inclusão, e nenhuma análise de poder foi feita devido à natureza observacional do estudo. A população do estudo foi dividida em três tercis para análise dos dados. As variáveis contínuas foram apresentadas como média ± desvio padrão (DP) ou mediana e intervalo interquartil (IQR), conforme apropriado. As variáveis categóricas são apresentadas por meio de frequências absolutas e relativas. Padrões de distribuição de variáveis contínuas e igualdade de variâncias entre tercis foram testados com testes de Shapiro-Wilk e Levene, respectivamente. Para variáveis contínuas, foram utilizados testes ANOVA unidirecional com correção de Welch ou testes de Kruskal-Wallis, dependendo da presença de padrão de distribuição normal. Análises post-hoc para ANOVA unidirecional foram feitas utilizando os testes HSD de Tukey ou Games-Howell, enquanto o teste Dwass-Steel-Critchlow-Fligner foi utilizado para análises feitas com o teste Kruskal-Wallis. Para variáveis categóricas, foi utilizado o teste qui-quadrado para comparações. Curvas de Kaplan-Meier foram traçadas para análise de sobrevida e os tercis individuais foram comparados com o teste log-rank. O modelo de riscos proporcionais de Cox foi utilizado para determinar preditores individuais de mortalidade em um ano. Todos os parâmetros com valor de p <0,10 na regressão univariada de Cox foram incluídos no modelo inicial, e um critério de seleção retroativo foi utilizado para construir o modelo final. Curvas receptor-operador foram traçadas para analisar a acurácia preditiva do ACEF-MDRD para a predição de mortalidade em um ano. Além disso, o teste de DeLong foi utilizado para determinar se o ACEF-MDRD não era inferior ao escore GWTG-HF em termos de precisão. O índice líquido de melhoria da reclassificação (NRI) foi calculado conforme descrito anteriormente. ^19^ Um valor de p <0,05 foi aceito como estatisticamente significativo para todas as análises. Todas as análises estatísticas foram feitas usando Jamovi (The Jamovi project - 2020). Jamovi versão 1.2 para Microsoft Windows), que é uma interface gráfica de usuário para linguagem R (R Core Team (2019). R: Uma linguagem e ambiente para computação estatística. Versão 3.6 para Microsoft Windows) e SPSS 25.0 (IBM Inc, Armonk, EUA).

Para evitar perda de dados na regressão de Cox e no teste de DeLong, um procedimento de imputação múltipla foi utilizado para prever valores faltantes. Um total de 5 imputações foram feitas, e os resultados de uma estimativa conjunta dessas 5 imputações foram apresentados como resultado sempre que possível. Para todos os outros testes estatísticos, foram utilizados dados originais e o número de casos em que os dados estavam disponíveis foi indicado entre parênteses. Como os dados sobre peptídeos natriuréticos eram muito escassos para serem imputados (> 50% dos dados estavam faltando), uma análise de subgrupo separada foi realizada para entender como a precisão prognóstica do escore ACEFMDRD foi comparada com a do peptídeo natriurético pró-tipo B N-terminal (NT-proBNP) em pacientes nos quais os dados estavam disponíveis.

## Resultados

A média de idade da população estudada foi de 63,7 ± 13,1 anos e 524 pacientes (70,1%) eram do sexo masculino. A mediana do escore ACEF-MDRD na população estudada foi de 2,43 (1,73 - 3,74) e a mediana do escore ACEF-MDRD foi de 1,51 (1,29 - 1,73), 2,41 (2,13 - 2,80) e 4,60 (3,74 - 5,77) para tercis ACEF-MDRD _baixo_ , ACEF-MDRD _med_ e ACEF-MDRD _alto_ , respectivamente. Cento e quarenta e dois pacientes (19,0%) faleceram ao final do acompanhamento de um ano.

**Figura 1:**
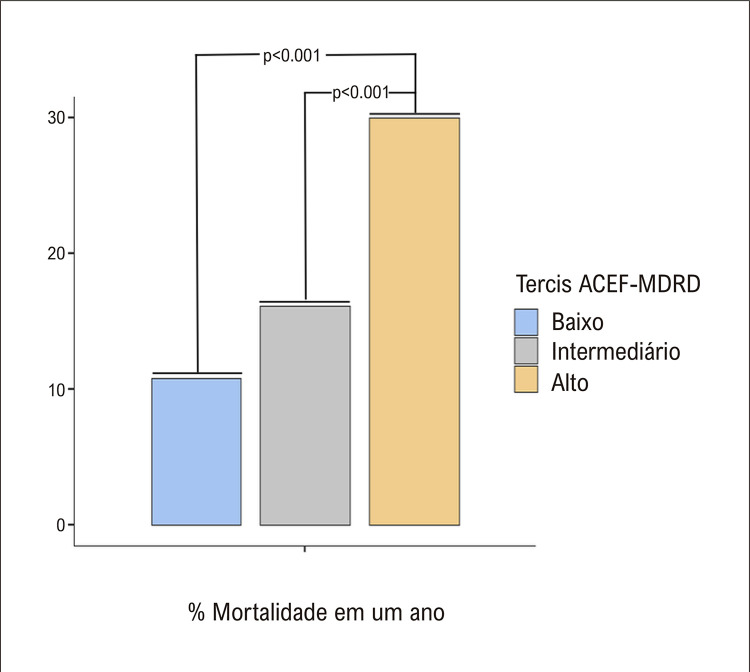
– Os gráficos de barras mostram a porcentagem de pacientes que morreram dentro de um ano de acompanhamento. As taxas médias de mortalidade previstas em um ano foram de 0,12, 0,16 e 0,29, respectivamente, para tercis de escore ACEF-MDRD baixo, intermediário e alto. ACEF-MDRD: Escore de Idade, Creatinina e Fração de Ejeção - Dieta Modificada em Doença Renal.

As características demográficas, antropométricas, clínicas e laboratoriais dos pacientes foram resumidas nas Tabelas 1 e 2. Como esperado, houve diferenças significativas entre os tercis em termos de características. Os pacientes dentro do tercil ACEF-MDRD _alto_ eram mais propensos a serem mais velhos e do sexo masculino do que o tercil ACEF-MDRD _baixo_ . Sintomas e sinais individuais de congestão e IC foram mais frequentes em pacientes dentro do tercil alto da ACEF-MDRD, e mais pacientes neste tercil apresentaram sintomas de classe 3 ou 4 da New York Heart Association (NYHA) em comparação com outros tercis. Além de apresentar maior creatinina e menor taxa de filtração glomerular no início do estudo, a hemoglobina e a albumina foram significativamente mais baixas, e o NT-proBNP foi significativamente maior no tercil alto do ACEF-MDRD. Por fim, tanto a frequência de pacientes com pelo menos uma internação quanto o número total de internações repetidas foram mais frequentes no tercil ACEF-MDRD _alto_ , e a mortalidade foi significativamente maior neste último tercil em comparação com ACEF-MDRD _med_ e ACEF-MDRD _baixo_ ( Figura 1 ).

**Tabela 1:** – Características antropométricas, demográficas e clínicas dos tercis da ACEF-MDRD

Características	ACEF-MDRD _**baixo**_ (n=249)	ACEF-MDRD _**med**_ (n=249)	ACEF-MDRD _**alto**_ (n=250)	Valor p
**Características demográficas**				
**Anos de idade)**	57,5±13,3	65,1±11,6***	68,6±11,7***	<0,001
**Sexo: Feminino)**	61 (24,5%)	62 (24,9%)	101 (40,4%)	<0,001
**Peso (kg) (n=624)**	79,1±14,9	76,3±14,6	74,8±14,2*	0,02
**Altura (cm) (n=620)**	167,0±8,22	167,0±8,34	165,0±8,45	0,11
**IMC (kg/m** ^**µ**^ **) (n=616)**	28,5±4,9	27,3±4,8*	27,4±4,7	0,01
**Características Clínicas**				
**Sinais vitais**				
PA sistólica (mmHg) (n=663)	120,0±18,3	121,0±17,9	119,0±19,8	0,49
PA diastólica (mmHg) (n=663)	73,8±10,3	73,2±11,2	74,1±12,1	0,81
Frequência cardíaca (batimentos/m) (n=657)	79,0±17,1	80,2±17,7	82,1±16,7*	0,04
**Tabagismo ativo (%)**	51 (2,05%)	37 (14,9%)	39 (15,6%)	0,19
**Diabetes (%)**	50 (20,1%)	75 (30,1%)	85 (34,0%)	0,002
**Hipertensão (ativa ou passada) (%)**	96 (38,6%)	112 (45,0%)	133 (53,2%)	0,004
**Doença pulmonar obstrutiva crônica (%)**	28 (11,2%)	39 (15,7%)	29 (11,6%)	0,261
**Infarto do miocárdio prévio (%)**	123 (49,4%)	122 (49,0%)	120 (48,0%)	0,95
**Revascularização Prévia**				
ICP (%)	96 (38,6%)	91 (36,5%)	93 (37,2%)	0,89
CRM (%)	47 (18,9%)	64 (25,7%)	51 (20,4%)	0,15
**Fibrilação Atrial (%) (n=672)**	57 (25,6%)	68 (29,8%)	58 (26,2%)	0,55
**Etiologia (n=666)**				
Cardiomiopatia Isquêmica (%)	134 (62,0%)	140 (61,9%)	143 (63,8%)	
Cardiomiopatia Dilatada/Outros (%)	82 (28,0%)	86 (38,1%)	81 (36,2%)	0,89
**Insuficiência Cardíaca de Novo (%)**	43 (17,3%)	59 (23,7%)	89 (35,6%)	<0,001
**Apresentação**				
Insuficiência Cardíaca Aguda (%)	67 (26,9%)	83 (33,3%)	116 (46,4%)	
Falha crônica do coração (%)	182 (73,1%)	166 (66,7%)	134 (53,6%)	<0,001
**Sintomas na apresentação**				
Dispneia aos esforços diários (%)	55 (22,1%)	69 (27,7%)	117 (48,8%)	<0,001
Dispneia paroxística	23 (9,2%)	29 (11,6%)	31 (12,4%)	0,50
Congestionamento autorrelatado (%)	39 (15,7%)	54 (21,7%)	105 (42,0%)	<0,001
Palpitações (%)	13 (5,2%)	15 (6,0%)	24 (9,6%)	0,12
**Resultados do exame**				
Distensão venosa jugular (%)	28 (11,2%)	72 (28,9%)	78 (31,2%)	<0,001
Edema Pré-tibial (%)	77 (30,9%)	89 (35,7%)	108 (43,2%)	0,02
Crepitações (%) (n=737)	58 (23,9%)	89 (35,9%)	136 (55,3%)	<0,001
**Aula da NYHA**				
NYHA 1 ou 2 (%)	160 (76,9%)	143 (63,3%)	93 (41,5%)	
NYHA 3 ou 4 (%)	48 (23,1%)	83 (36,7%)	131 (58,5%)	<0,001
**Dispositivos Cardíacos Implantáveis**				
Marcapasso VVI (%)	15 (5,6%)	10 (4,0%)	16 (6,4%)	0,48
Marcapasso DDD (%)	9 (3,6%)	8 (3,2%)	11 (4,4%)	0,77
CDI (%)	35 (14,1%)	52 (20,9%)	49 (19,6%)	0,11
Ressincronização Cardíaca (%)	5 (2,0%)	15 (6,0%)	18 (7,2%)	0,02

Valores de p abaixo de 0,05 foram apresentados em negrito. IMC: índice de massa corporal; PA: pressão arterial; CRM: cirurgia de revascularização do miocárdio; CDI: desfibrilador cardioversor implantável; NYHA: New York Heart Association; ICP: intervenção coronária percutânea. *valor de p<0,05 comparado ao tercil ACEF-MDRD _baixo._ ** valor de p <0,01 <0,05 comparado ao tercil ACEF-MDRD _baixo._ *** valor p <0,001 <0,05 comparado ao tercil ACEF-MDRD _baixo._

**Tabela 2:** – Valores laboratoriais, medicamentos e resultados para tercis ACEF-MDRD

Característica	ACEF-MDRD _**baixo**_ (n=249)	ACEF-MDRD _**med**_ (n=249)	ACEF-MDRD _**alto**_ (n=250)	Valor p
**Características laboratoriais**				
Hemoglobina (g/dl) (n=738)	13,5±2,01	13,2±1,77	12,2±1,98***	<0,001
Nitrogênio ureico no sangue (n=621)	21,0±11,1	28,1±14,7***	40,2±21,1***	<0,001
Creatinina	0,90±0,16	1,00±0,23**	1,71±0,81***	<0,001
TFG-MDRD	91,8±23,7	79,7±23,5***	46,2±21,5***	<0,001
BNP (n=44)	27,9 (20,4-64,2)	70,7 (33,3-116,0)	30,3 (21,5-40,9)	0,09
NT-proBNP (n=211)	941,0 (498,0-2660,0)	1537,0 (634,0-4850,0)	2798,0 (560,0-5310,0) *	0,03
Sódio (n=739)	138,0 ± 4,0	138,0±3,9	137,0±6,0	0,06
Albumina (n=426)	3,94±0,60	3,92±0,69	3,74±0,70***	<0,001
**Medicamentos**				
Inibidores da ECA (%)	171 (68,7%)	162 (65,1%)	160 (64,0%)	0,51
Bloqueadores dos receptores da angiotensina (%)	82 (32,9%)	84 (33,7%)	101 (40,4%)	0,16
Bloqueadores beta (%)	224 (90,0%)	229 (92,0%)	229 (91,6%)	0,70
Bloqueadores dos receptores mineralocorticoides (%)	149 (59,8%)	160 (64,3%)	140 (56,0%)	0,17
Diuréticos (%)	96 (38,6%)	114 (46,0%)	113 (46,3%)	0,14
Digoxina (%)	22 (8,8%)	39 (15,7%)	28 (11,5%)	0,06
**Resultados**				
Pelo menos uma internação durante o acompanhamento (%) (n=670)	112 (51,1%)	137 (60,1%)	151 (67,7%)***	0,002
Número total de internações durante o acompanhamento (%) (n=668)	1,00 (0,00-1,00)	1,00 (0,00-2,00)	1,00 (0,00-2,25)***	<0,001
Mortalidade por todas as causas (%)	27 (10,8%)	40 (16,1%)	75 (30,0%)	<0,001

Valores de p abaixo de 0,05 foram apresentados em negrito. BNP: peptídeo natriurético tipo B, TFG-MDRD: taxa de filtração glomerular calculada com a fórmula Dieta Modificada em Doença Renal, NT-proBNP: N-terminal do pró-peptídeo natriurético tipo B. *valor de p<0,05 comparado ao tercil ACEF-MDRD _baixo._ ** valor de p <0,01 <0,05 comparado ao tercil ACEF-MDRD _baixo._ *** valor p <0,001 <0,05 comparado ao tercil ACEF-MDRD _baixo._

**Figura 2:**
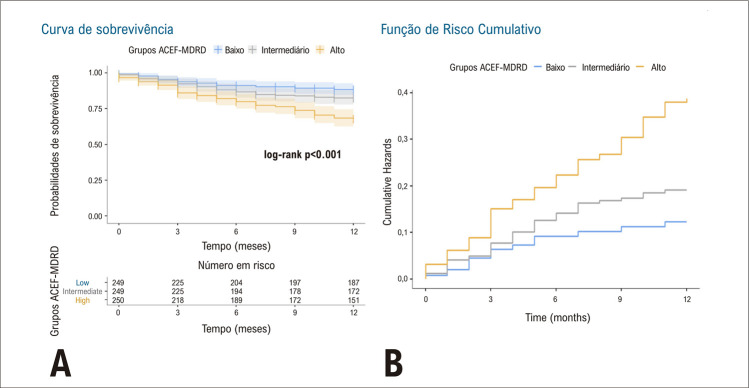
Curva de Kaplan-Meier para sobrevida em um ano (A) e taxa de risco cumulativo (B) para tercis ACEF-MDRD. As áreas coloridas ao redor das linhas sólidas indicam intervalos de confiança. ACEF-MDRD: Escore de Idade, Creatinina e Fração de Ejeção - Dieta Modificada em Doença Renal.

As curvas de Kaplan-Meier para sobrevida em um ano e riscos cumulativos para tercis foram fornecidas na Figura 2 . Houve diferenças significativas entre os tercis ACEF-MDRD em termos de sobrevida em um ano. Em comparações pareadas, os pacientes no tercil ACEF _alto_ tiveram sobrevida em um ano significativamente menor do que o tercil ACEF-MDRD _baixo_ e ACEF-MDRD _med_ (p<0,001). Houve também uma tendência de menor sobrevida no tercil ACEF-MDRD _med_ em comparação ao tercil ACEF-MDRD _baixo_ , mas isso não foi estatisticamente significativo (p=0,08).

Os preditores univariados e multivariados de mortalidade foram fornecidos na Tabela 3 . Após ajuste, houve uma relação linear entre cada aumento de um ponto no escore ACEF-MDRD e a mortalidade em um ano. Além do ACEF-MDRD, outros parâmetros associados à mortalidade foram a presença de congestão autorreferida na admissão, menor sódio e maior classe NYHA.

**Tabela 3:** – Preditores univariados e multivariados de mortalidade em um ano

Característica	Análise Univariada		Análise multivariada	

	OU (IC 95%)	Valor p	OU (IC 95%)	Valor p
Apresentação (IC aguda)	3,56 (2,54 – 5,00)	<0,001	2,26 (1,55 – 3,29)	<0,001
Congestionamento auto-relatado (presença de)	2,95 (2,12 – 4,10)	<0,001		
Dispneia (presença de)	2,43 (1,75 – 3,38)	<0,001		
Frequência cardíaca (aumento por batimentos/minuto)	1,01 (1,00 – 1,02)	0,02		
Dispneia paroxística noturna (presença de)	2,02 (1,34 – 3,05)	0,001		
Distensão jugular (presença de)	2,10 (1,50 – 2,96)	<0,001		
Edema pré-tibial (presença de)	1,50 (1,08 – 2,09)	0,02		
Crepitações (presença de)	3,12 (2,21 – 4,39)	<0,001		
Hemoglobina (aumento por g/dl)	0,84 (0,78 – 0,91)	<0,001		
Sódio (aumento por g/dl)	0,96 (0,94 – 0,98)	<0,001	0,97 (0,95 – 0,99)	0,013
NYHA (Classe 3/4)	4,02 (2,77 – 5,82)	<0,001	2,45 (1,60 – 3,72)	<0,001
ACEF-MDRD (por aumento de 1 ponto)	1,28 (1,17 – 1,38)	<0,001	1,14 (1,04 – 1,24)	0,006

Todas as variáveis que tiveram valor de p <0,1 foram fornecidas na tabela. As variáveis do modelo final foram fornecidas nas colunas relevantes. NYHA: New York Heart Association.

ACEF-MDRD apresentou estatística-c global de 0,66 ± 0,03 para predição de mortalidade em um ano e, para ponto de corte de 2,71, teve sensibilidade de 71,1%, especificidade de 61,9%, valor preditivo positivo de 30,1%. e valor preditivo negativo de 90,1%. Todas as variáveis componentes do ACEF-MDRD tiveram uma estatística C mais baixa para prever a mortalidade em um ano em comparação com o ACEF-MDRD (idade: 0,62 ± 0,03, fração de ejeção do ventrículo esquerdo: 0,64 ± 0,03, taxa de filtração glomerular: 0,56 ± 0,03, taxa de filtração glomerular: 0,56 ± 0,03, p=0,001).

**Figura 3:**
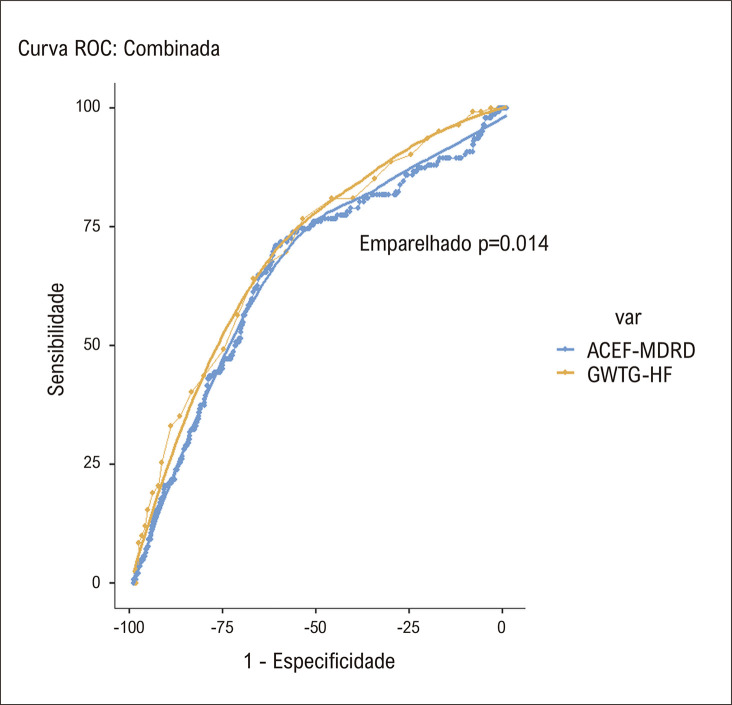
Curvas receptor-operador para modelos ACEF-MDRD e GWTG-HF para predição de mortalidade em um ano na população estudada. As linhas interrompidas mostram curvas reais, enquanto as linhas sólidas mostram suavização LOESS para comparação de dois modelos. ACEF-MDRD: Idade, Creatinina e Fração de Ejeção - Escore de Dieta Modificada em Doença Renal, GWTG-HF: Siga as Diretrizes - Escore de Insuficiência Cardíaca.

Em um modelo de regressão multivariada composto pelos escores ACEF-MDRD e GWTG-HF ambos foram encontrados como preditores independentes de mortalidade em um ano (OR:1,08 (IC95%:1,05 - 1,11), p<0,001 para escore GWTG-HF e OR:1,12 (IC95%: 1,02 - 1,23), p=0,02 para ACEF-MDRD). Para prever a mortalidade em um ano, o escore GWTG-HF apresentou estatística c de 0,70 ± 0,02, e a diferença entre o escore GWTG-HF e o ACEF-MDRD não foi estatisticamente diferente ( Figura 3 ). No geral, o NRI foi de 0,107, indicando uma melhoria na previsão de mortalidade com o escore ACEF-MDRD em relação ao escore GWTG-HF. Os componentes individuais das análises do NRI mostraram que a previsão correta da mortalidade em um ano foi ligeiramente inferior com o ACEF-MDRD (NRIe -0,023), mas a previsão da sobrevida em um ano foi muito melhor quando o ACEF-MDRD foi usado (NRIne 0,130).

No subgrupo de pacientes nos quais um NT-proBNP estava disponível (n = 211, 28,2% da amostra do estudo), o NT-proBNP foi significativamente maior em pacientes que morreram ao final de um ano em comparação com aqueles que sobreviveram ( 2510 (390 - 4994) pg/ml vs. 1399 (547 - 4113) pg/ml, p<0,001). Comparado ao NT-proBNP, a capacidade preditiva do escore ACEF-MDRD foi significativamente maior ( Figura 1 suplementar). O escore ACEF-MDRD permaneceu um preditor significativo de mortalidade em um ano após ajuste para NT-proBNP neste subgrupo (OR: 1,45, IC 95%: 1,22 - 1,73, p<0,001).

## Discussão

Tal como muitas outras doenças médicas, o prognóstico de um determinado paciente com IC tem uma natureza estocástica – e não determinística. Como resultado direto, um modelo de risco nunca poderia ter uma capacidade discriminatória perfeita para a mortalidade, independentemente da complexidade do modelo. Usar muitas variáveis para um modelo de risco torna-o menos útil para a prática clínica e aumenta o risco de “ *overfitting* ” – o que ameaça a precisão de um modelo quando aplicado a populações diferentes da amostra de derivação original. ^20^ De preferência, um modelo deve seguir o “lei da parcimônia” e contém o menor número de variáveis com maior valor, em vez de incluir todas as variáveis que fornecem apenas um aumento marginal na precisão. O presente estudo mostrou que um escore de risco simples composto apenas por três variáveis tem boa precisão preditiva para mortalidade em um ano e apresenta desempenho bastante comparável a escores de risco mais complexos, como o modelo GWTG-HF. As principais conclusões do presente trabalho estão resumidas na Figura Central .

Os modelos de risco têm desvantagens importantes que limitam a sua utilidade. Um modelo de risco de IC poderia fornecer resultados imprecisos quando aplicado a populações além da sua derivação inicial; eles raramente são precisos na previsão do prognóstico para pacientes com IC individuais e podem se tornar obsoletos com o tempo. ^21 , 22^ No entanto, ainda são convenientes, pois os modelos de risco permitem uma avaliação mais objetiva da esperança média de vida e podem ser úteis para selecionar a estratégia de gestão ideal para um determinado paciente com IC. ^21 , 22^ Mesmo modelos de risco com validação externa são subutilizados na prática clínica diária, talvez pelas limitações e pela inconveniência de encontrar e inserir múltiplos dados para calcular o escore final. ^23^ Escore de risco MAGGIC, que tem uma boa base de evidências para validade e um formidável escore c de 0,74 para mortalidade quando aplicado a outras coortes de IC, precisa de 13 variáveis diferentes para serem inseridas. ^24^ O escore GWTG-HF teve uma capacidade preditiva aceitável para mortalidade em um ano (c- o escore variou entre 0,64 - 0,67 para ICFEr e ICFEp, respectivamente), embora fossem necessárias apenas 7 variáveis que tornassem o escore GWTG-HF um pouco mais fácil de calcular e mais compatível com a lei da parcimônia. ^25^ Os resultados atuais indicam que o escore ACEF-MDRD poderia prever a mortalidade em um ano com uma precisão comparável ao escore GWTG e, semelhante ao escore GWTG-HF, poderia ser aplicado a populações com IC, independentemente do fenótipo apresentado. O escore ACEF-MDRD teve a vantagem adicional de usar três parâmetros simples e universalmente disponíveis que tornam seu cálculo conveniente, tornando-o um pouco mais adequado para ir além do “domínio da pesquisa” para o mundo real do que outros modelos de risco.

Os componentes do escore ACEF não são usados apenas como preditores independentes de prognóstico na IC, mas também uma ou mais dessas variáveis são comumente encontradas em quase todos os escores de risco de IC. ^3 , 4 , 16 , 26^ A combinação dessas variáveis permite uma estimativa global do expectativa de vida, comorbidades, função de órgãos-alvo e desempenho ventricular esquerdo. Apesar da disponibilidade de múltiplos estudos que demonstram a capacidade preditiva do escore ACEF em diversas condições cardiovasculares, incluindo pacientes com infarto do miocárdio recente ou aqueles submetidos a cirurgia cardiovascular ou intervenções percutâneas, os dados sobre a utilidade prognóstica do escore ACEF em pacientes com IC são extremamente limitados. ^8 - 12^ Chen et al. estudaram ACEF e ACEF-MDRD em 862 pacientes com cardiomiopatia isquêmica e descobriram que ambos os escores tinham uma boa capacidade discriminativa (a estatística C foi de 0,73 para ACEF e 0,72 para ACEF-MDRD, respectivamente). No entanto, não ficou claro se estes pacientes tinham IC concomitante, uma vez que este estudo foi apresentado apenas como um resumo. ^27^ Os resultados atuais sugerem que o escore ACEF-MDRD é um preditor independente de mortalidade em todos os pacientes com IC, independentemente da etiologia subjacente, apresentação, ou fenótipo, tornando-se assim uma ferramenta potencialmente útil para vários pacientes.

Note-se que o escore ACEF-MDRD não foi desenvolvida a partir da presente amostra, mas aplicada a ela e, como tal, a presente análise em si deve ser considerada um estudo de validação. Embora muitos estudos tenham relatado uma precisão preditiva mais impressionante para seus modelos do que os números fornecidos neste estudo, eles carecem de validação externa ou sua precisão preditiva é substancialmente menor quando testados em amostras diferentes de suas coortes de derivação. ^28^ Dado que as estatísticas C fornecidas raramente excede 0,8 para quase todos os modelos, o uso de um índice com uma precisão preditiva bastante modesta poderia ser justificado dada a simplicidade do cálculo (que poderia ser feito até mesmo com caneta e papel), tornando-o prático para o uso diário e a falta de “ *overfitting* ” - tornando-o adequado para uso em diferentes populações de IC. ^22^

Os tratamentos disponíveis para a IC são numerosos na era contemporânea e os algoritmos fornecidos para orientar as estratégias de tratamento não são baseados em evidências. Embora a principal expectativa de um modelo de risco seja uma estimativa da mortalidade global, é, no entanto, mais útil quando pode orientar as decisões de tratamento. Vários estudos mostraram que modelos de risco poderiam ser utilizados para esse fim. Por exemplo, foi demonstrado que o Modelo de Insuficiência Cardíaca de Seattle (SHFM) prediz a mortalidade após o implante de dispositivo de assistência ventricular esquerda. ^29^ Se o escore ACEF-MDRD poderia ser utilizado de forma semelhante seria uma perspectiva interessante para pesquisa em estudos futuros.

Os presentes resultados indicam que o escore ACEF-MDRD teve uma capacidade discriminativa bastante modesta para mortalidade. Adicionar novas variáveis à equação seria uma forma de melhorar a precisão, uma vez que nossos achados indicam que o escore ACEF por si só não explica toda a variabilidade na mortalidade. No entanto, esta abordagem violaria o princípio fundador do escore ACEF, que utilizava um número limitado de preditores em vez de todas as variáveis com significância estatística na análise multivariada. Outra forma seria encontrar preditores de mortalidade semelhantes, porém mais poderosos, para redesenhar o escore ACEF-MDRD. Embora os componentes individuais do escore ACEF sejam preditores independentes de mortalidade, não está claro se são os melhores preditores, uma vez que o escore ACEF não foi desenvolvido para prever a mortalidade após IC. Como tal, melhores preditores poderiam ser usados para substituir os componentes principais do escore ACEF, mas a lei da parcimônia ainda deve ser aplicada para manter os preditores no mínimo.

### Limitações do estudo

Apesar do desenho multicêntrico do estudo, o número de pacientes inscritos foi bastante limitado, afetando assim o poder da análise. Algumas variáveis estavam faltando e precisavam ser imputadas para análises multivariadas. Os dados faltantes foram superiores a 50% para algumas variáveis, e esses parâmetros - principalmente os peptídeos natriuréticos - não puderam ser incluídos nas análises multivariadas. Embora o ACEF-MDRD parecesse ter um significado prognóstico independente no subgrupo de 211 pacientes nos quais as concentrações de NT-proBNP estavam disponíveis, esta análise foi invariavelmente tendenciosa devido ao pequeno tamanho da amostra e à disponibilidade de dados de alguns centros. Assim, uma amostra maior é necessária para determinar se o escore ACEF-MDRD tem utilidade adicional em relação aos peptídeos natriuréticos. Da mesma forma, escores preditivos como o escore MAGGIC ou o Modelo de Insuficiência Cardíaca de Seattle não puderam ser calculados devido à falta de dados, portanto a utilidade do ACEF-MDRD em relação a essas ferramentas permanece incerta. Finalmente, embora os presentes resultados forneçam uma verificação externa para o escore ACEF-MDRD, mais dados de estudos adicionais aumentariam a confiabilidade para uso clínico futuro do escore ACEF-MDRD em pacientes com IC.

## Conclusões

O escore ACEF-MDRD é um preditor independente de mortalidade em um ano em pacientes com insuficiência cardíaca e sua acurácia preditiva é comparável à do escore GWTG-HF. Em contraste com outros modelos “complexos” que necessitam de múltiplas variáveis e ferramentas especializadas para cálculo, o ACEF-MDRD necessita de três variáveis simples para estimativa de mortalidade, tornando-se uma alternativa bastante mais conveniente para a prática clínica diária.
