# Supplementary material for: Utilidade da Idade, Creatinina e Fração de Ejeção - Modificação da Dieta no Escore de Doença Renal para Prever a Sobrevivência em Pacientes com Insuficiência Cardíaca
Source: Arq Bras Cardiol. 2023 Dec 14;120(12):e20230158. [Article in Portuguese] doi: 10.36660/abc.20230158 (PMC10789375; doi:10.36660/abc.20230158)
Supplement: Translation

# Translation: Usefulness of Age, Creatinine and Ejection Fraction - Modification of Diet in Renal Disease Score for Predicting Survival in Patients with Heart Failure

**Authors:** Rengin Çetin Güvenç, Tolga Sinan Güvenç, Yüksel Çavuşoğlu, Ahmet Temizhan, Mehmet Birhan Yılmaz

**Affiliations:** 1 Turkey Okan University Faculty of Medicine, Department of Cardiology, Istanbul – Turkey; 2 Turkey Istinye University Faculty of Medicine, Department of Cardiology, Istanbul – Turkey; 3 Turkey Eskisehir Osmangazi University, Department of Cardiology, Eskisehir – Turkey; 4 Turkey Ankara City Hospital, Department of Cardiology, Ankara – Turkey; 5 Turkey Dokuz Eylul University Faculty of Medicine, Department of Cardiology, Izmir – Turkey

**Keywords:** Heart Failure, Mortality, Survivorship

## Abstract

**Background:**

While many risk models have been developed to predict prognosis in heart failure (HF), these models are rarely useful for the clinical practitioner as they include multiple variables that might be time-consuming to obtain, they are usually difficult to calculate, and they may suffer from statistical overfitting.

**Objectives:**

To investigate whether a simpler model, namely the ACEF-MDRD score, could be used for predicting one-year mortality in HF patients.

**Methods:**

748 cases within the SELFIE-HF registry had complete data to calculate the ACEF-MDRD score. Patients were grouped into tertiles for analyses. For all tests, a p-value <0.05 was accepted as significant.

**Results:**

Significantly more patients within the ACEF-MDRD _high_ tertile (30.0%) died within one year, as compared to other tertiles (10.8% and 16.1%, respectively, for ACEF-MDRD _low_ and ACEF-MDRD _med_ , p<0.001 for both comparisons). There was a stepwise decrease in one-year survival as the ACEF-MDRD score increased (log-rank p<0.001). ACEF-MDRD was an independent predictor of survival after adjusting for other variables (OR: 1.14, 95%CI:1.04 – 1.24, p=0.006). ACEF-MDRD score offered similar accuracy to the GWTG-HF score for predicting one-year mortality (p=0.14).

**Conclusions:**

ACEF-MDRD is a predictor of mortality in patients with HF, and its usefulness is comparable to similar yet more complicated models.

## Introduction

It has been estimated that at least 23 million people have heart failure (HF), making it one of the most common cardiovascular disorders in the contemporary age. ^1^ Despite the advances in the screening, diagnosis, and management of HF, mortality rates remain high, with a rate of 121 per 1000 patient-years for patients with preserved ejection fraction (HFpEF) and 141 per 1000 patients for patients with a reduced ejection fraction (HFrEF). ^2^ While clinical judgment and individual parameters are commonly employed for prognostication, multiple risk models are also available to estimate mortality and to guide management decisions. ^3 - 7^

A common issue with these risk models is that they generally suffer from “overfitting” of multiple redundant variables that are not useful in estimating prognosis in other HF cohorts where the mortality rate differs from the original derivation cohort. ^8^ Moreover, the necessity of using numerous (and sometimes laborious to obtain) variables to calculate a single risk score for each HF patient usually renders these scores impractical for clinical use in a busy clinic.

Age, creatinine, and ejection fraction (ACEF) score was initially developed to predict postoperative mortality after cardiovascular surgery while keeping the “law of parsimony” in mind. ^8^ However, later studies have found the ACEF score or its simple modifications computed by substituting creatinine with glomerular filtration rate (GFR) with Modified Diet in Renal Disease (MDRD) equation - the ACEF-MDRD score - were useful to predict mortality or complications following percutaneous coronary or structural interventions, as well as those who had acute coronary syndromes. ^9 - 12^ Individual variables used to calculate the ACEF score have already been shown as predictors of hospitalizations and mortality in patients with HF, and it is reasonable to consider that a score calculated using these variables would have better usefulness in predicting mortality in HF. ^13 - 16^ In the present analysis, we sought to investigate whether the ACEF-MDRD score could predict one-year mortality in HF patients and to understand how the ACEF-MDRD score compares to other established but more complex models, such as the Get With The Guidelines - Heart Failure (GWTG-HF) score.

## Methods

The design and execution of the SELFIE-TR registry have been published before. ^17^ To summarize, 23 study centers representing all geographic areas in Turkey were included in the SELFIE-TR study. The diagnosis of HF was established using a combination of clinical evaluation, echocardiographic, and laboratory findings, and the diagnosis was independently confirmed by at least two cardiologists working at each study center. All patients 18 years old or older and accepted enrolment to the study were included; no exclusion criteria were used. One thousand fifty-four patients were enrolled, and one-year survival data became recently available for 1022 out of these 1054 patients. ^18^ Of these patients, 748 had complete data to calculate the ACEF-MDRD score, and all analyses were done using these records.

All patients in the SELFIE-TR registry gave their informed consent before inclusion, and the present study was conducted according to the principles outlined in the 1975 Declaration of Helsinki and its revisions. The study was approved by an ethics committee (approval no 288-AU/003), and regulatory approval was obtained in each study center per laws and other regulations.

All laboratory measurements were done at the individual centers, and samples used for analyses were withdrawn soon after the inclusion of the patient in the study. Not all measurements were available for all patients due to the differences between the centers regarding local resources. The glomerular filtration rate was calculated using the MDRD equation. Ejection fraction was measured with two-dimensional echocardiography in each study center with modified Simpson’s method by two cardiologists blinded to each other’s measurement, and an average of these two measurements was taken as the final result. ACEF-MDRD score was calculated as follows:

Age/ejection fraction + 1 point per every 10 mL/min reduction in GFR when GRF was below 60 ml/m ^2^ /min.

### Statistical analyses

The sample size was determined by the number of cases eligible for inclusion, and no power analyses were done due to the study’s observational nature. The study population was divided into three tertiles for data analysis. Continuous variables were given as mean ± standard deviation (SD) or median and interquartile range (IQR) as appropriate. Categorical variables are presented through absolute and relative frequencies. Patterns of distribution of continuous variables and equality of variances across tertiles were tested with Shapiro-Wilk and Levene tests, respectively. For continuous variables, either a one-way ANOVA test with Welch correction or Kruskal-Wallis tests were used depending on the presence of a normal distribution pattern. Post-hoc analyses for one-way ANOVA were done using Tukey’s HSD or Games-Howell tests, while the Dwass-Steel-Critchlow-Fligner test was used for analyses done with the Kruskal-Wallis test. For categorical variables, the chi-square test was used for comparisons. Kaplan-Meier curves were drawn for survival analysis, and individual tertiles were compared with the log-rank test. Cox proportional hazards model was used to determine individual predictors of one-year mortality. All parameters with a p-value <0.10 on univariate Cox regression were included in the initial model, and a backward selection criterion was used to construct the final model. Receiver-operator curves were drawn to analyze the predictive accuracy of ACEF-MDRD for the prediction of one-year mortality. Additionally, DeLong’s test was used to determine whether ACEF-MDRD was non-inferior to the GWTG-HF score in terms of accuracy. The net reclassification improvement index (NRI) was calculated as described before. ^19^ A p-value of <0.05 was accepted as statistically significant for all analyses. All statistical analyses were done using Jamovi (The Jamovi project (2020). Jamovi version 1.2 for Microsoft Windows), which is a graphical user interface for R language (R Core Team (2019). R: A Language and environment for statistical computing. Version 3.6 for Microsoft Windows) and SPSS 25.0 (IBM Inc, Armonk, USA).

To avoid data loss in Cox regression and DeLong’s test, a multiple imputation procedure was used to predict missing values. A total of 5 imputations were done, and results from a pooled estimate of these 5 imputations were given as the result whenever possible. For all other statistical tests, original data was used, and the number of cases in whom data was available was indicated in parentheses. Since the data on natriuretic peptides was too scarce to be imputed (>50% of data was missing), a separate subgroup analysis was performed to understand how the prognostic accuracy of the ACEF-MDRD score compared to that of N-terminal pro-B-type natriuretic peptide (NT-proBNP) in patients in whom the data was available.

## Results

The mean age of the study population was 63.7 ± 13.1 years, and 524 patients (70.1%) were male. The median ACEF-MDRD score in the study population was 2.43 (1.73 - 3.74), and median ACEF-MDRD scores were 1.51 (1.29 - 1.73), 2.41 (2.13 - 2.80) and 4.60 (3.74 - 5.77) for ACEF-MDRD _low_ , ACEF-MDRD _med_ and ACEF-MDRD _high_ tertiles, respectively. One hundred forty-two patients (19.0%) were dead at the end of the one-year follow-up.

The patients’ demographic, anthropometric, clinical, and laboratory characteristics were summarized in Tables 1 and 2. As expected, there were significant differences across tertiles in terms of characteristics. Patients within the ACEF-MDRD _high_ tertile were likelier to be older and male than ACEF-MDRD _low_ tertile. Individual symptoms and signs of congestion and HF were more frequent in patients within the ACEF-MDRD _high_ tertile, and more patients in this tertile had New York Heart Association (NYHA) class 3 or 4 symptoms compared to other tertiles. Besides having a higher creatinine and lower glomerular filtration rate at baseline, hemoglobin and albumin were significantly lower, and NT-proBNP was significantly higher in ACEF-MDRD _high_ tertile. Finally, both the frequency of patients with at least one hospitalization and the total number of repeat hospitalizations were more frequent in the ACEF-MDRD _high_ tertile, and mortality was significantly higher in the latter tertile compared to both ACEF-MDRD _med_ and ACEF-MDRD _low_ ( Figure 1 ).

**Table 1:** – Anthropometric, demographic, and clinical characteristics of ACEF-MDRD tertiles

Characteristics	ACEF-MDRD _**low**_ (n=249)	ACEF-MDRD _**med**_ (n=249)	ACEF-MDRD _**high**_ (n=250)	p-value
**Demographic Characteristics**				
**Age (years)**	57.5±13.3	65.1±11.6***	68.6±11.7***	<0.001
**Gender (%Female)**	61 (24.5%)	62 (24.9%)	101 (40.4%)	<0.001
**Weight (kg) (n=624)**	79.1±14.9	76.3±14.6	74.8±14.2*	0.02
**Height (cm) (n=620)**	167.0±8.22	167.0±8.34	165.0±8.45	0.11
**BMI (kg/m2) (n=616)**	28.5±4.9	27.3±4.8*	27.4±4.7	0.01
**Clinical Characteristics**				
**Vital Signs**				
Systolic BP (mmHg) (n=663)	120.0±18.3	121.0±17.9	119.0±19.8	0.49
Diastolic BP (mmHg) (n=663)	73.8±10.3	73.2±11.2	74.1±12.1	0.81
Heart rate (beats/m) (n=657)	79.0±17.1	80.2±17.7	82.1±16.7*	0.04
**Active smoking (%)**	51 (2.05%)	37 (14.9%)	39 (15.6%)	0.19
**Diabetes (%)**	50 (20.1%)	75 (30.1%)	85 (34.0%)	0.002
**Hypertension (active or past) (%)**	96 (38.6%)	112 (45.0%)	133 (53.2%)	0.004
**Chronic Obstructive Pulmonary Disease (%)**	28 (11.2%)	39 (15.7%)	29 (11.6%)	0.261
**Previous Myocardial Infarction (%)**	123 (49.4%)	122 (49.0%)	120 (48.0%)	0.95
**Previous Revascularization**				
PCI (%)	96 (38.6%)	91 (36.5%)	93 (37.2%)	0.89
CABG (%)	47 (18.9%)	64 (25.7%)	51 (20.4%)	0.15
**Atrial Fibrillation (%) (n=672)**	57 (25.6%)	68 (29.8%)	58 (26.2%)	0.55
**Etiology (n=666)**				
Ischemic Cardiomyopathy (%)	134 (62.0%)	140 (61.9%)	143 (63.8%)	
Dilated Cardiomyopathy/Other (%)	82 (28.0%)	86 (38.1%)	81 (36.2%)	0.89
**De Novo Heart Failure (%)**	43 (17.3%)	59 (23.7%)	89 (35.6%)	<0.001
**Presentation**				
Acute Heart Failure (%)	67 (26.9%)	83 (33.3%)	116 (46.4%)	
Chronic Heart Failure (%)	182 (73.1%)	166 (66.7%)	134 (53.6%)	<0.001
**Symptoms at presentation**				
Dyspnea on daily exertion (%)	55 (22.1%)	69 (27.7%)	117 (48.8%)	<0.001
Paroxysmal dyspnea	23 (9.2%)	29 (11.6%)	31 (12.4%)	0.50
Self-reported congestion (%)	39 (15.7%)	54 (21.7%)	105 (42.0%)	<0.001
Palpitations (%)	13 (5.2%)	15 (6.0%)	24 (9.6%)	0.12
**Examination Findings**				
Jugular Venous Distention (%)	28 (11.2%)	72 (28.9%)	78 (31.2%)	<0.001
Pretibial Edema (%)	77 (30.9%)	89 (35.7%)	108 (43.2%)	0.02
Crepitations (%) (n=737)	58 (23.9%)	89 (35.9%)	136 (55.3%)	<0.001
**NYHA Class**				
NYHA 1 or 2 (%)	160 (76.9%)	143 (63.3%)	93 (41.5%)	
NYHA 3 or 4 (%)	48 (23.1%)	83 (36.7%)	131 (58.5%)	<0.001
**Cardiac Implantable Devices**				
VVI Pacemaker (%)	15 (5.6%)	10 (4.0%)	16 (6.4%)	0.48
DDD Pacemaker (%)	9 (3.6%)	8 (3.2%)	11 (4.4%)	0.77
ICD (%)	35 (14.1%)	52 (20.9%)	49 (19.6%)	0.11
Cardiac Resynchronization (%)	5 (2.0%)	15 (6.0%)	18 (7.2%)	0.02

P-values below 0.05 were given in bold. BMI: Body mass index, BP: Blood pressure, CABG: Coronary artery bypass grafting, ICD: Implantable cardioverter defibrillator, NYHA: New York Heart Association, PCI: Percutaneous coronary intervention. * p-value <0.05 compared to ACEF-MDRD _low_ tertile; ** p-value <0.01 <0.05 compared to ACEF-MDRD _low_ tertile; *** p-value <0.001 <0.05 compared to ACEF-MDRD _low_ tertile.

**Table 2:** – Laboratory values, medications, and outcomes for ACEF-MDRD tertiles

Characteristic	ACEF-MDRD _**low**_ (n=249)	ACEF-MDRD _**med**_ (n=249)	ACEF-MDRD _**high**_ (n=250)	p value
**Laboratory characteristics**				
Hemoglobin (g/dl) (n=738)	13.5±2.01	13.2±1.77	12.2±1.98***	<0.001
Blood urea nitrogen (n=621)	21.0±11.1	28.1±14.7***	40.2±21.1***	<0.001
Creatinine	0.90±0.16	1.00±0.23**	1.71±0.81***	<0.001
GFR-MDRD	91.8±23.7	79.7±23.5***	46.2±21.5***	<0.001
BNP (n=44)	27.9 (20.4-64.2)	70.7 (33.3-116.0)	30.3 (21.5-40.9)	0.09
NT-proBNP (n=211)	941.0 (498.0-2660.0)	1537.0 (634.0-4850.0)	2798.0 (560.0-5310.0) *	0.03
Sodium (n=739)	138.0 ± 4.0	138.0±3.9	137.0±6.0	0.06
Albumin (n=426)	3.94±0.60	3.92±0.69	3.74±0.70***	<0.001
**Medications**				
ACE inhibitors (%)	171 (68.7%)	162 (65.1%)	160 (64.0%)	0.51
Angiotensin receptor blockers (%)	82 (32.9%)	84 (33.7%)	101 (40.4%)	0.16
Beta-blockers (%)	224 (90.0%)	229 (92.0%)	229 (91.6%)	0.70
Mineralocorticoid receptor blockers (%)	149 (59.8%)	160 (64.3%)	140 (56.0%)	0.17
Diuretics (%)	96 (38.6%)	114 (46.0%)	113 (46.3%)	0.14
Digoxin (%)	22 (8.8%)	39 (15.7%)	28 (11.5%)	0.06
**Outcomes**				
At least one hospitalization during follow-up (%) (n=670)	112 (51.1%)	137 (60.1%)	151 (67.7%)***	0.002
Total number of hospitalizations during follow-up (%) (n=668)	1.00 (0.00-1.00)	1.00 (0.00-2.00)	1.00 (0.00-2.25)***	<0.001
All-cause mortality (%)	27 (10.8%)	40 (16.1%)	75 (30.0%)	<0.001

P-values below 0.05 were given in bold. BNP: B-type natriuretic peptide, GFR-MDRD: Glomerular filtration rate calculated with Modified Diet in Renal Disease formula, NT-proBNP: N-terminal of the pro-B-type natriuretic peptide. * p-value <0.05 compared to ACEF-MDRD _low_ tertile; ** p-value <0.01 <0.05 compared to ACEF-MDRD _low_ tertile; *** p-value <0.001 <0.05 compared to ACEF-MDRD _low_ tertile.

**Figure 1:** – Bar graphs show the percentage of patients who died within one year of follow-up. Predicted mean one-year mortality rates were 0.12, 0.16, and 0.29, respectively, for low, intermediate, and high ACEF-MDRD score tertiles. ACEF-MDRD: Age, Creatinine, and Ejection Fraction - Modified Diet in Renal Disease score.

Kaplan-Meier curves for one-year survival and cumulative hazards for tertiles were provided in Figure 2 . There were significant differences between the ACEF-MDRD tertiles in terms of one-year survival. On pairwise comparisons, patients within the ACEF _high_ tertile had significantly lower one-year survival than ACEF-MDRD _low_ and ACEF-MDRD _med_ tertile (p<0.001). There was also a trend towards lower survival in the ACEF-MDRD _med_ tertile compared to ACEF-MDRD _low_ tertile, but this was not statistically significant (p=0.08).

**Figure 2:** – Kaplan-Meier Curve for one-year survival (A) and cumulative hazard ratio (B) for ACEF-MDRD tertiles. Colored areas around the solid lines indicate confidence intervals. ACEF-MDRD: Age, Creatinine, and Ejection Fraction - Modified Diet in Renal Disease score.

Univariate and multivariate predictors of mortality were provided in Table 3 . After adjustment, there was a linear relationship between each one-point increase in the ACEF-MDRD score and one-year mortality. In addition to ACEF-MDRD, other parameters associated with mortality were the presence of self-reported congestion at admission, lower sodium, and higher NYHA class.

**Table 3:** – Univariate and multivariate predictors of one-year mortality

Characteristic	Univariate Analysis		Multivariate Analysis	

	OR (95% CI)	p-value	OR (95% CI)	p-value
Presentation (Acute HF)	3.56 (2.54 – 5.00)	<0.001	2.26 (1.55 – 3.29)	<0.001
Self-reported congestion (presence of)	2.95 (2.12 – 4.10)	<0.001		
Dyspnea (presence of)	2.43 (1.75 – 3.38)	<0.001		
Heart rate (per beats/minute increase)	1.01 (1.00 – 1.02)	0.02		
Paroxysmal nocturnal dyspnea (presence of)	2.02 (1.34 – 3.05)	0.001		
Jugular distention (presence of)	2.10 (1.50 – 2.96)	<0.001		
Pretibial edema (presence of)	1.50 (1.08 – 2.09)	0.02		
Crepitations (presence of)	3.12 (2.21 – 4.39)	<0.001		
Hemoglobin (per g/dl increase)	0.84 (0.78 – 0.91)	<0.001		
Sodium (per g/dl increase)	0.96 (0.94 – 0.98)	<0.001	0.97 (0.95 – 0.99)	0.013
NYHA (Class 3/4)	4.02 (2.77 – 5.82)	<0.001	2.45 (1.60 – 3.72)	<0.001
ACEF-MDRD (per 1 point increase)	1.28 (1.17 – 1.38)	<0.001	1.14 (1.04 – 1.24)	0.006

All variables that had a p-value <0.1 were provided in the table. Variables in the final model were provided in the relevant columns. NYHA: New York Heart Association.

ACEF-MDRD had an overall c-statistic of 0.66 ± 0.03 for prediction of one-year mortality, and for a cut-off point of 2.71, it had a sensitivity of 71.1%, specificity of 61.9%, positive predictive value of 30.1% and negative predictive value of 90.1%. All component variables of ACEF-MDRD had a lower c-statistic for predicting one-year mortality as compared to ACEF-MDRD (age: 0.62 ± 0.03, left ventricular ejection fraction: 0.64 ± 0.03, glomerular filtration rate: 0.56 ± 0.03, overall p=0.001).

On a multivariate regression model consisting of ACEF-MDRD and GWTG-HF scores, both were found as independent predictors of one-year mortality (OR:1.08 (95%CI:1.05 - 1.11), p<0.001 for GWTG-HF score and OR:1.12 (95%CI: 1.02 - 1.23), p=0.02 for ACEF-MDRD). For predicting one-year mortality, the GWTG-HF score had a c-statistic of 0.70 ± 0.02, and the difference between the GWTG-HF score and ACEF-MDRD was not statistically different ( Figure 3 ). Overall, NRI was 0.107, indicating an improvement in mortality prediction with ACEF-MDRD score over GWTG-HF score. Individual components of the NRI analyses have shown that the correct prediction of one-year mortality was slightly inferior with ACEF-MDRD (NRIe -0.023), but the prediction of one-year survival was much better when ACEF-MDRD was used (NRIne 0.130).

**Figure 3:** – Receiver-operator curves for ACEF-MDRD and GWTG-HF models for predicting one-year mortality in the study population. Interrupted lines show actual curves, while solid lines show LOESS smoothing for comparison of two models. ACEF-MDRD: Age, Creatinine and Ejection Fraction - Modified Diet in Renal Disease score, GWTG-HF: Get With The Guidelines - Heart Failure score.

In the subgroup of patients in whom an NT-proBNP was available (n=211, 28.2% of the study sample), NT-proBNP was significantly higher in patients who were dead at the end of one year as compared to those who survived (2510 (390 - 4994) pg/ml vs. 1399 (547 - 4113) pg/ml, p<0.001). Compared to NT-proBNP, the predictive ability of the ACEF-MDRD score was significantly higher (Supplementary Figure 1 ). ACEF-MDRD score remained a significant predictor of one-year mortality after adjustment for NT-proBNP in this subgroup (OR:1.45, 95%CI: 1.22 - 1.73, p<0.001).

## Discussion

Like many other medical disorders, the prognosis of a particular patient with HF has a stochastic - rather than deterministic - nature. As a direct result, a risk model could never have a perfect discriminatory ability for mortality, regardless of the complexity of the model. Using too many variables for a risk model makes it less useful for clinical practice and increases the risk of ‘overfitting’ - which threatens the accuracy of a model when applied to populations other than the original derivation sample. ^20^ Preferably, a model should follow the “law of parsimony” and contain the least number of variables with the most value rather than including every variable that only provides a marginal increase in accuracy. The present study showed that a simple risk score consisting only of three variables has good predictive accuracy for one-year mortality and performs rather comparably to more complex risk scores such as the GWTG-HF model. The main findings of the present work are summarized in the Central Illustration .

Risk models have important drawbacks that limit their usefulness. An HF risk model could give inaccurate results when applied to populations beyond their initial derivation; they are rarely accurate in predicting prognosis for individual HF patients and can become obsolete with time. ^21 , 22^ However, they are still convenient as risk models enable a more objective assessment of the average life expectancy, and they could be useful for selecting the optimal management strategy for a given HF patient. ^21 , 22^ Even risk models with external validation are underutilized in daily clinical practice, perhaps because of the limitations and the inconvenience of finding and entering multiple data to calculate the final score. ^23^ MAGGIC risk score, which has a good evidence base for validity and a formidable c-score of 0.74 for mortality when applied to other HF cohorts, needs 13 different variables to be entered. ^24^ GWTG-HF score had an acceptable predictive ability for one-year mortality (c-score varied between 0.64 - 0.67 for HFrEF and HFpEF, respectively), though it needed a mere 7 variables that made GWTG-HF score somewhat easier to calculate and more compatible with the law of parsimony. ^25^ Present findings indicate that the ACEF-MDRD score could predict one-year mortality with an accuracy comparable to the GWTG score; and similar to the GWTG-HF score, it could be applied to HF populations regardless of the presenting phenotype. ACEF-MDRD score had the additional advantage of using three simple and universally available parameters that make it convenient to calculate, thus making it somewhat better suited to move beyond the “research realm” to the real world than other risk models.

The components of the ACEF score are not only used as standalone predictors of prognosis in HF but also one or more of these variables are commonly found in nearly all HF risk scores. ^3 , 4 , 16 , 26^ Combining these variables allows an overall estimation of life expectancy, comorbidities, end-organ function, and left ventricular performance. Despite the availability of multiple studies demonstrating the predictive ability of ACEF score in many different cardiovascular conditions, including patients with recent myocardial infarction or those undergoing cardiovascular surgery or percutaneous interventions, data on the prognostic usefulness of ACEF score in patients with HF is extremely limited. ^8 - 12^ Chen et al. have studied ACEF and ACEF-MDRD in 862 patients with ischemic cardiomyopathy and found that both scores had a good discriminative ability (c-statistics were 0.73 for ACEF and 0.72 for ACEF-MDRD, respectively). However, whether these patients had accompanying HF was unclear, as this study was only presented as an abstract. ^27^ Present findings suggest that the ACEF-MDRD score is an independent predictor of mortality in all HF patients, regardless of the underlying etiology, presentation, or phenotype, thus making it a potentially useful tool for various patients.

To note, the ACEF-MDRD score was not developed from the present sample but applied to it, and as such, the present analysis itself should be considered a validation study. While many studies have reported a more impressive predictive accuracy for their models than the figures provided in this study, they either lack external validation or their predictive accuracy is substantially lower when tested in samples other than their derivation cohorts. ^28^ Given that provided c-statistics rarely exceed 0.8 for nearly all models, using an index with a rather modest predictive accuracy could be justified given the sheer simplicity of the calculation (which could be done even with a pen and paper) making it practical for daily use and the lack of “overfitting” - making it suitable for use in different HF populations. ^22^

Available treatments for HF are numerous in the contemporary era, and algorithms provided to guide management strategies are not evidence-based. While the main expectation from a risk model is an estimation of overall mortality, it is nonetheless more useful when it can guide treatment decisions. Several studies have shown that risk models could be utilized for this aim. For example, the Seattle Heart Failure Model (SHFM) has been shown to predict mortality after left ventricular assist device implantation. ^29^ Whether the ACEF-MDRD score could be utilized similarly would be an interesting prospect to research in future studies.

Present findings indicate that the ACEF-MDRD score had a rather modest discriminative ability for mortality. Adding new variables to the equation would be one way to improve the accuracy since our findings indicate that the ACEF score itself does not explain all the variability in mortality. However, this approach would violate the founding principle of the ACEF score, which was using a limited number of predictors rather than every variable with statistical significance in multivariate analysis. Another way would be to find similar yet more powerful predictors of mortality to redesign the ACEF-MDRD score. Although individual components of the ACEF score are standalone predictors of mortality, it is unclear whether they are the best predictors, as the ACEF score was not developed to predict mortality after HF. As such, better predictors could be used to replace core components of the ACEF score, but the law of parsimony should still be applied to keep the predictors at a minimum.

### Study limitations

Despite the multicenter design of the study, the number of patients enrolled was rather limited, thus affecting the power of the analysis. Some variables were missing and needed to be imputed for multivariate analyses. The missing data was higher than 50% for some variables, and these parameters - most notably natriuretic peptides - could not be included in the multivariate analyses. Although ACEF-MDRD appeared to have an independent prognostic significance in the subgroup of 211 patients in whom NT-proBNP concentrations were available, this analysis was invariably biased due to the small sample size and data availability from a few centers. Thus, a larger sample is needed to determine whether the ACEF-MDRD score has additional usefulness over natriuretic peptides. Similarly, predictive scores such as the MAGGIC score or Seattle Heart Failure Model could not be calculated due to missing data, so the usefulness of ACEF-MDRD over these tools remains uncertain. Finally, while present findings provide an external verification for the ACEF-MDRD score, more data from additional studies would increase the reliability for future clinical use of ACEF-MDRD score in HF patients.

## Conclusions

ACEF-MDRD score is an independent predictor of one-year mortality in patients with heart failure, and its predictive accuracy is comparable to the GWTG-HF score. In contrast to other “complex” models needing multiple variables and specialized tools for calculation, ACEF-MDRD needs three simple variables for mortality estimation, making it a rather more convenient alternative for daily clinical practice.
